# Comparative outcomes of coil embolization and surgical clipping in elderly patients with subarachnoid hemorrhage: a systematic review and meta-analysis

**DOI:** 10.1007/s10143-025-03713-9

**Published:** 2025-08-04

**Authors:** Yohanna Idsabella Rossi, Gabriel Bolner, Jonathan Costa Dall’Acqua, Fabiana Dolovitsch de Oliveira, Lucas Vincenzi Zacaria, Taís Luise Denicol, Michel Frudit, Natália Vasconcellos De Oliveira Souza

**Affiliations:** 1 Federal University of Health Sciences of Porto Alegre, Porto Alegre, Rio Grande do Sul Brazil; 2 Intervention Neuroradiology and Neurosurgery Department, University of São Paulo, São Paulo, Brazil; 3 Albert Einstein Hospital, Neurology and Intervention Neuroradiology Department, Av. Albert Einstein, 627/701 - Morumbi, São Paulo, SP 05652-900 Brasil; 4 Department of Neurology, Thomas Jefferson University Hospital, Philadelphia, PA USA

**Keywords:** Subarachnoid hemorrhage, Stroke, Elderly, Coiling, Endovascular, Microsurgery, Clipping

## Abstract

**Background and objectives:**

Elderly patients with subarachnoid hemorrhage (SAH) face a disproportionately high burden of morbidity and mortality. While endovascular coiling is often favored in this population, direct comparisons with surgical clipping are limited. We conducted a meta-analysis to compare outcomes of clipping versus coiling in SAH patients aged ≥ 60 years.

**Methods:**

A systematic search of PubMed, Embase, and Cochrane databases identified studies comparing the two treatments in this age group. The primary outcome was a composite of unfavorable outcomes (modified Rankin Scale [mRS] > 2 and mortality). Secondary outcomes included mortality, favorable outcome (mRS 0–2), rebleeding, and hospital length of stay. Heterogeneity was assessed using I² statistics, with subgroup analysis by age decade.

**Results:**

Twenty-seven studies (2 randomized controlled trials [RCTs]) involving 51,415 patients (59.6% treated with clipping) were included. There were no significant differences between clipping and coiling for unfavorable outcome (RR 1.03; 95% CI 0.96–1.11), favorable outcome (RR 1.02; 95% CI 0.93–1.11), mortality (RR 1.08; 95% CI 0.97–1.19), or rebleeding (RR 1.21; 95% CI 0.57–2.57). However, coiling was associated with shorter hospital stays (MD -2.53 days; 95% CI -4.58 to -0.49; *p* = 0.0152). RCTs showed a non-significant trend favoring coiling, while observational studies leaned toward clipping. Heterogeneity for main outcomes was moderate (I² = 57.7%). Using the GRADE framework, overall certainty of evidence was rated very low, mainly due to the predominance of non-randomized studies, moderate risk of bias, and inconsistency across studies.

**Conclusions:**

In SAH patients aged ≥ 60 years, clipping and coiling show comparable outcomes, with coiling associated with shorter hospital stays. Given the very low certainty of evidence, these findings should be interpreted with caution. Prospective multicenter cohorts are needed to establish more definitive evidence.

**Supplementary Information:**

The online version contains supplementary material available at 10.1007/s10143-025-03713-9.

## Introduction

Aneurysmal subarachnoid hemorrhage (SAH) accounts for only 5–10% of all strokes, yet carries a disproportionate burden of morbidity and mortality, resulting in significant loss of productive life years and societal costs [1, 2]. Despite advancements in diagnosis and treatment over the past few decades, which have led to lower rates of rebleeding, around 25% of patients die before reaching the hospital [3]. Among survivors, 30–40% experience poor clinical outcomes, often due to complications like delayed cerebral ischemia, hydrocephalus, and infections. Advanced age further exacerbates these risks, and elderly patients experience higher readmission rates and 30-day mortality [4–6]. While endovascular coiling is often preferred in this population, robust comparative evidence guiding the choice between surgical clipping and coiling remains insufficient [7].

For ruptured aneurysms, early endovascular coiling has been shown to improve survival without disability at one year compared to surgical clipping [8]. Long-term follow-up reveals this benefit persists, as the endovascular group maintains significantly lower probabilities of death or dependency at ten years [9]. Experts and small randomized trials agree that endovascular treatment is a more reasonable option in elderly patients due to its less traumatic nature and faster recovery, however, data from retrospective studies suggest comparable outcomes between clipping and coiling, with no significant difference in mortality, rehabilitation discharge rates, or readmission compared to coiling [10–13].

In response, we conducted a meta-analysis comparing clipping versus coil embolization for the treatment of aneurysmal SAH specifically in elderly patients (≥ 60 years). Additionally, subgroup analysis will be performed for each decade after the age of 60.

## Methods

This protocol was registered in the International Prospective Register of Systematic Reviews (PROSPERO) (CRD42024582126), and this study was conducted in accordance with the Cochrane Handbook for Systematic Reviews of Interventions, adhering to PRISMA (Preferred Reporting Items for Systematic Review and Meta-Analysis) [14, 15].

### Inclusion and exclusion criteria’s

We included randomized controlled trials (RCTs) and observational studies that directly compared the outcomes of coiling versus clipping for aneurysmal SAH, provided they: (1) reported outcomes specifically for elderly patients (≥ 60 years), either through subgroup analyses or dedicated cohorts, and (2) offered comparative data between both interventions. We imposed no restrictions on publication date, language, or follow-up duration. We excluded studies that: (1) lacked direct coiling-clipping comparisons (single-arm studies), (2) didn’t analyze elderly patients (≥ 60 years), (3) had overlapping populations with other included studies, (4) included ≤ 5 elderly patients with SAH, (5) consisted of case reports, case series, letters to the editor, comments, or editorials, or 4) focused on traumatic, perimesencephalic, or included nonaneurismatic SAH.

### PICO criteria

The PICOTS framework (Population, Intervention, Comparison, Outcome, Timing, and Setting) was used to define the review question:

#### Population (P)

Elderly patients (> 60 years) with aneurysmal subarachnoid hemorrhage (SAH) who underwent clipping or coiling, with studies providing subgroup analysis for this age group.

#### Intervention (I)

Endovascular coiling as the primary treatment.

#### Comparison (C)

Surgical clipping as the control treatment.

**Outcomes (O)**:

Primary outcome: Compound unfavorable outcome defined as modified Rankin Scale (mRS) score > 2 or mortality when mRS was unavailable. Since this approach may introduce bias, we also included a favorable outcome with only the mRS results. Studies that presented the Glasgow Outcome Scale (GOS) had their results converted to the mRS to allow simpler analyses, using conversion methods already described in the literature [16].

Secondary outcomes: all-cause mortality, favorable outcome (mRS ≤ 2), rebleeding rate (confirmed radiographically), and hospital length of stay (days).

#### Timing (T)

Outcomes assessed at hospital discharge and at the last available clinical follow-up (minimum 3 months post-SAH).

#### Setting (S)

Tertiary care hospitals or specialized centers.

### Search strategy

We systematically searched PubMed, Embase, and the Cochrane Library for studies published from inception to 5 August 2024, using the keywords: “subarachnoid hemorrhage”, “coiling”, “clipping”, “elderly”, and their variations (Supplemental Digital Content 1, Search strategy). Furthermore, we manually searched all potentially reference lists of the eligible studies. All references were exported to Rayyan QCRI and deduplicated [17]. The titles/abstracts and full-text articles were independently screened by two authors (JCD and FDO) and discrepancies were resolved by a third author.

### Data extraction

Two independent reviewers (GB, LZ) extracted data using a standardized sheet, including: study details (authors, year, country, design), sample size, patient demographics (age, sex), number of aneurysms, ruptured aneurysm location, baseline mRS, World Federation of Neurological Surgeons (WFNS) grade, modified Fisher score (mFs), follow-up duration. Discrepancies were resolved by a third investigator (YIR). To ensure the inclusion of studies that might otherwise be excluded and to minimize potential bias, non-English studies were translated using Google Translate, a free and accessible automated translation tool [18]. The accuracy of this tool has been previously validated against manual translation, demonstrating acceptable reliability [19].

### Data analysis

The statistical analysis was conducted using the ‘meta’ package in R statistical software (version 4.3.2). The longest available follow-up data was selected for outcomes with multiple timepoints. To address the limited number of RCTs for certain interventions, we combined data from both observational and RCTs, as recommended by the Cochrane’s Handbook [20]. A random effects meta-analysis was performed using the restricted maximum likelihood model to account for potential heterogeneity between RCTs and observational studies. For binary outcomes, the Mantel-Haenszel method was used to calculate the Risk Ratios (RR) with 95% confidence intervals (CI). When only the odds ratio was reported in a given study, we opted to use this measure of association for the outcome. For continuous outcomes, the means and standard deviations (SD) were used to calculate the Mean Difference (MD) and corresponding 95% CI. Statistical significance was set at *p* < 0.05. We assessed heterogeneity using Cochran’s Q test (*p* < 0.1) and I^2^ statistics (> 40%) [20]. Results were presented in forest plots. Publication bias was evaluated via Egger’s test alongside funnel plot for outcomes with 10 or more studies, as these tests lose statistical power when applied to fewer than 10 studies [20]. To ensure the robustness of the findings, leave-one-out sensitivity analysis was performed for primary and secondary outcomes. Subgroup analyses were based on age (60–69, 70–79, ≥ 80 years), study design (RCT vs. observational), and follow-up time duration. Additionally, subgroup analyses including only studies that adjusted their estimates were performed whenever possible.

### Quality assessment and confidence of the evidence

Two independent reviewers (JCD and LZ) assessed risk of bias using the Newcastle Ottawa Scale (NOS) for observational studies, and the Revised Cochrane risk-of-bias tool (RoB 2) for RCTs [21, 22]. The NOS evaluates studies across three domains —selection, comparability, and outcome—using eight items, resulting in an overall risk of bias assessment [22]. The RoB 2 tool assesses risk of bias across five domains: randomization, deviation from intended interventions, measurement of outcomes, missing outcome data and selection of reported results. Based on this, studies were classified as having a low risk of bias, some concerns, or a high risk of bias [21]. Discrepancies were resolved by consensus.

Two reviewers (TLD/GB) evaluated the certainty of the evidence using GRADE (Grading of Recommendations Assessment, Development, and Evaluation) system [23], classifying evidence as “very low,” “low,” “moderate,” or “high”. Factors that may decrease the quality of evidence include inconsistency of results, risk of bias, indirectness, imprecision, and publication bias. Conversely, factors as a large magnitude of effect, dose-response gradient, and effect of plausible residual confounding may increase the quality of evidence. All disagreements were resolved through consensus.

## Results

### Study characteristics

**Fig. 1:**
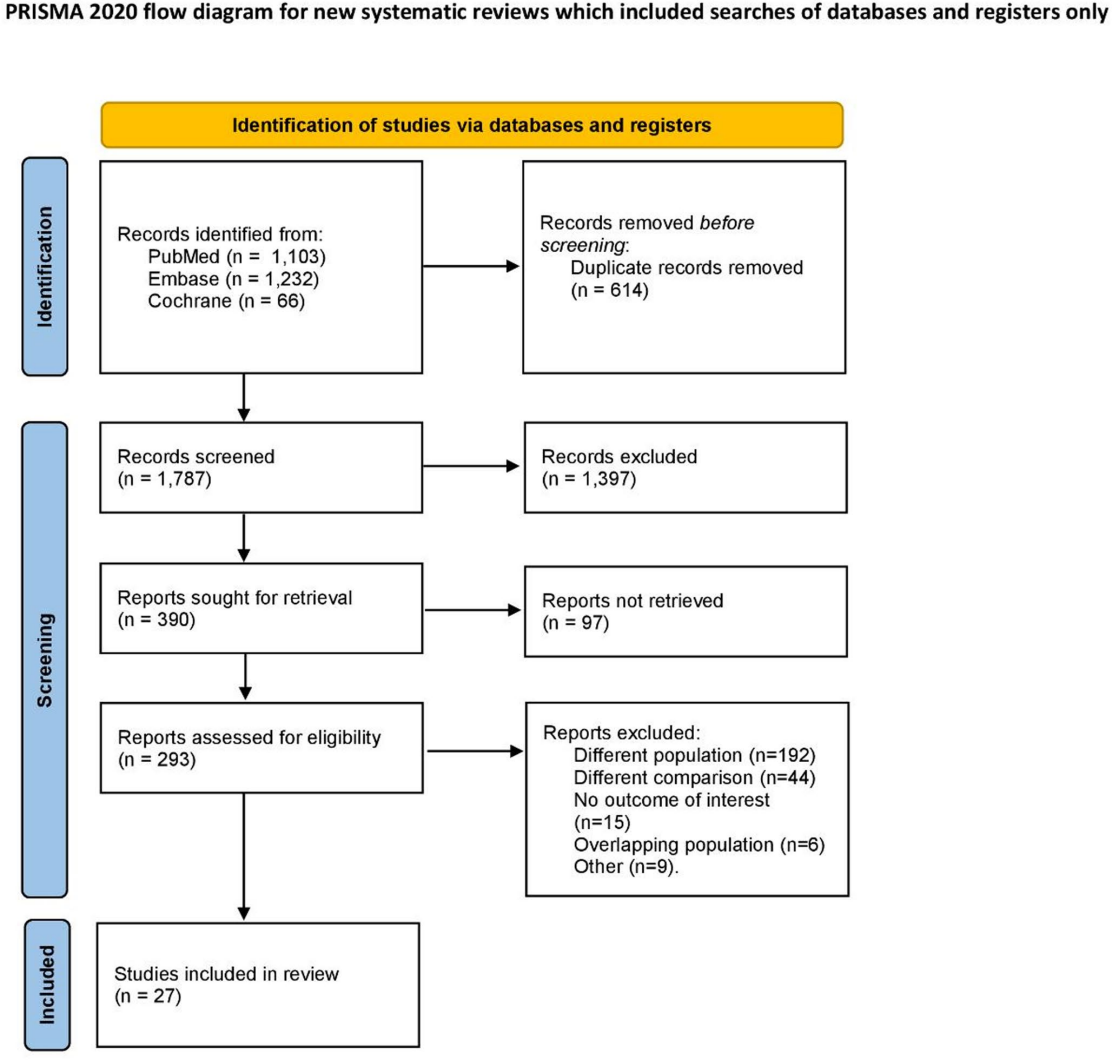
PRISMA flowchart of the article selection process. PRISMA, Preferred Reporting Items for Systematic Reviews and Meta-Analyses

The electronic search strategy yielded 2,401 results. After removing duplicates and screening titles and abstracts, 390 publications were deemed relevant and underwent full-text review. Following the screening process, 27 studies were included in the analysis (Fig. 1). Among these, 2 were RCTs [24, 25], 4 were prospective cohorts [13, 26–28], and the remaining studies were retrospective or retrospective/prospective cohorts [11, 12, 29–47].

The ISAT (International Subarachnoid Aneurysm Trial) trial had two published reports with relevant data for elderly patients [8]: the primary report (used for the main analysis) [25], and a secondary report (providing mortality data for patients ≥ 65 years) [48], as this information was not available in the original publication. Data from Proust et al. (2018, 2019) were treated separately as they analyzed randomized and non-randomized patients, respectively [13, 24]. Follow-up duration ranged from discharge to 180 months (median = 12, IQR = 17.5). Complete study characteristics are presented in Table 1.

**Table 1:** Baseline characteristics

First Author, year	Country	Study design	Follow-up (months)	Clipping / Embolization or coiling	Total sample size	Age, mean	Age cutoff	Female (%)	Posterior circulation (%)
**Groden et al.**,** 2000**	Germany	Observational	180	5/3	8	69.2	60	N/A	N/A
**Groden et al.**,** 2001**	Germany	Observational	26	7/6	13	69.5	60	69.2	N/A
**Molyneux et al.**,** 2005**	Europe	RCT	48	258/259	2,143*	N/A	60	63*	2.7*
**Braun et al.**,** 2005**	Germany	Observational	16	18/17	35	N/A	65	N/A	8.6
**Nieuwkamp et al.**,** 2006**	Netherlands	Observational	4	34/13	139*	N/A	75	82*	8*
**Asano et al.**,** 2007**	Japan	Observational	6	5/1	6	81	80	66.6	0
**Karamanakos et al.**,** 2010**	Finland	Observational	12	96/49	145	N/A	70	80	7.6
**Proust et al.**,** 2010**	France	Observational	6	34/30	64	N/A	70	76.5	11
**Tenjin et al.**,** 2011**	Japan	Observational	2	14/22	113*	N/A	70	N/A	4.4*
**Schöller et al.**,** 2012**	Germany	Observational	154	123/93	256*	68	60	66*	20*
**Shirao et al.**,** 2012**	Japan	Observational	0	118/38	156	74.5	65	73.7	13.5
**Brinjikji et al.**,** 2013**	USA	Observational	0	10,166/9,139	19,305	N/A	65	N/A	N/A
**Park et al.**,** 2014**	South Korea	Observational	0	46/62	159*	71.6	65	84.2*	10.4*
**Park et al. (critical age)**,** 2014**	South Korea	Observational	12	85/80	165	75.1 (± 4.3)**	70	86.3	8.5
**Bekelis et al.**,** 2015**	USA	Observational	12	1,206/2,004	3,210	N/A	65	74.8	N/A
**Kutsuna et al.**,** 2017**	Japan	Observational	0	3/2	5	91.5 (± 1.7)**	90	80	60
**Wang et al.**,** 2017**	China	Observational	98	7/2	9	65.5	60	66.6	0
**Dasenbrock et al.**,** 2018**	USA	Observational	24	288/1,010	1,298	84.7 (± 3.5)**	80	82	N/A
**Proust et al.**,** 2018**	France	RCT	12	21/20	41	N/A	70	75.6	0
**Zheng et al.**,** 2018**	China	Observational	12	34/49	83	N/A	60	65	9.6
**Proust et al.**,** 2019**	France	Observational	12	54/208	262	77.9 (± 5.1)**	70	82.4	10
**Hironaka et al.**,** 2020**	Japan	Observational	0	17,740/7,422	25,162	N/A	61	69.2	26.8
**Maeda et al.**,** 2020**	Japan	Observational	0	85/39	124	81.2 (± 4.6)**	75	81.2	15.4
**Yoshikawa et al.**,** 2020**	Japan	Observational	12	64/54	118	N/A	70	75.4	18.7
**Catapano et al.**,** 2021**	USA	Observational	72	53/24	77	71.4 (± 4.8)**	65	N/A	15
**Hovorka et al.**,** 2023**	Czech Republic	Observational	3	92/38	130	N/A	65	N/A	N/A
**Lee et al.**,** 2024**	Singapore	Observational	6	50/84	134	68.5	60	77.6	23.1

* Specific data for elderly patients was not provided. The findings are derived from the overall study population

** Standard deviation

### Patients’ characteristics

A total of 51,415 participants were included, of whom 30,685 were treated with surgical clipping. Among studies presenting baseline characteristics, 70% (23893/33970) were female [11, 13, 24–29, 32, 34, 35, 37–44, 46, 47], and 24% (7062/29396) had a ruptured posterior circulation aneurysm [12, 13, 24–29, 31, 35, 37–44, 46, 47].

### Risk of bias assessment results

The RoB2 assessment identified “some concerns” regarding the risk of bias in the two RCTs, particularly related to the randomization process, as blinding results assessment was not feasible due to the nature of the interventions (surgical clips and endovascular coils are easily distinguishable), Supplemental Digital Content 1, Table S2 [24, 25]. For the observational studies, the NOS tool classified 20 studies into the “Moderate risk of bias” category [11–13, 27–29, 32, 33, 35–42, 44, 46, 47, 49] and the remaining in the “Low risk of bias” category, Supplemental Digital Content 1, Table S3 [26, 31, 34, 43]. No studies were suspected of having a high risk of bias.

### Primary outcome (unfavorable outcome, mRS > 2 and mortality)

**Fig. 2:**
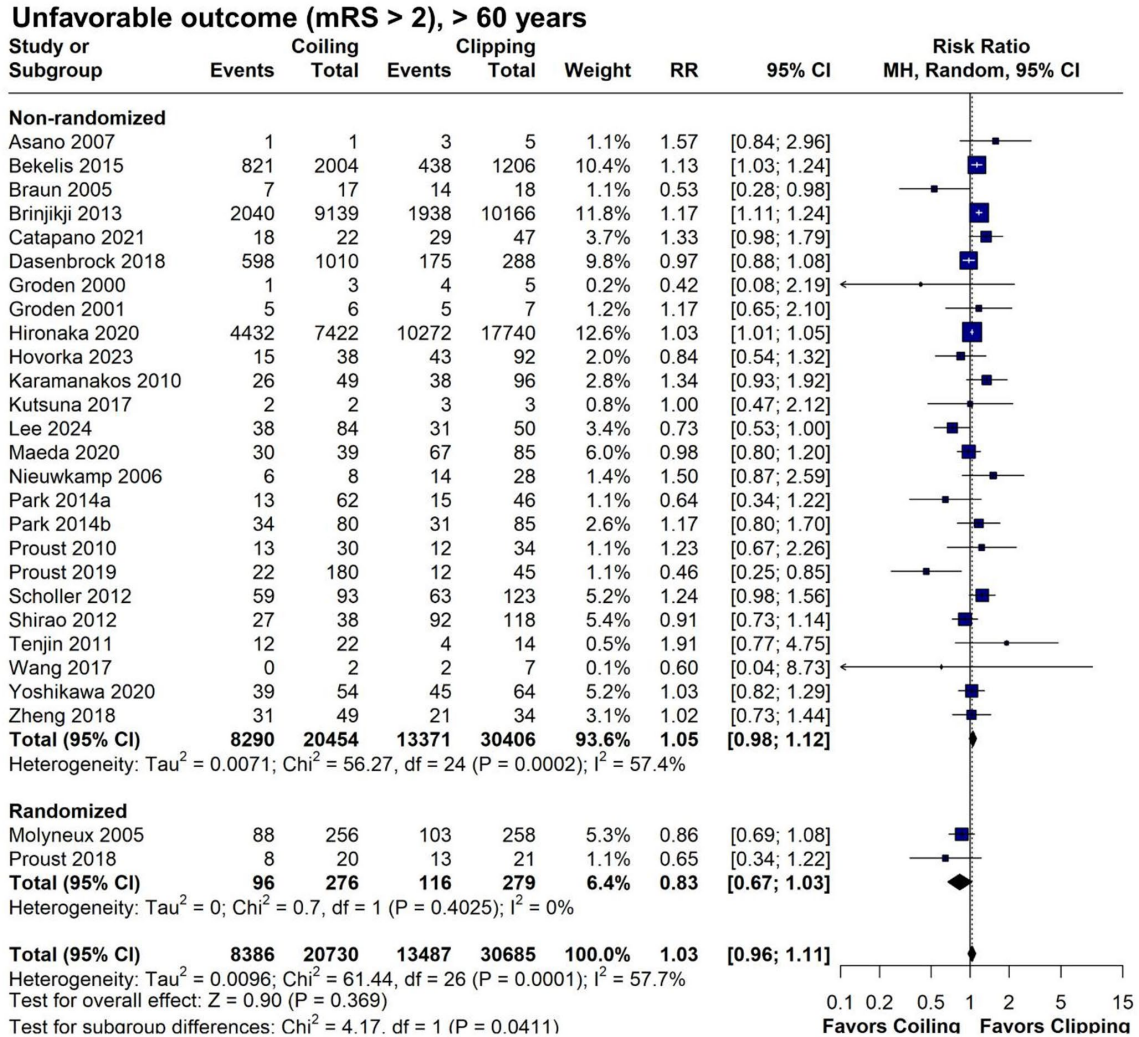
Forest plot comparing coiling versus clipping for unfavorable outcome (mRS > 2) in patients ≥ 60 years old. RR, risk ratio

**Fig. 3:**
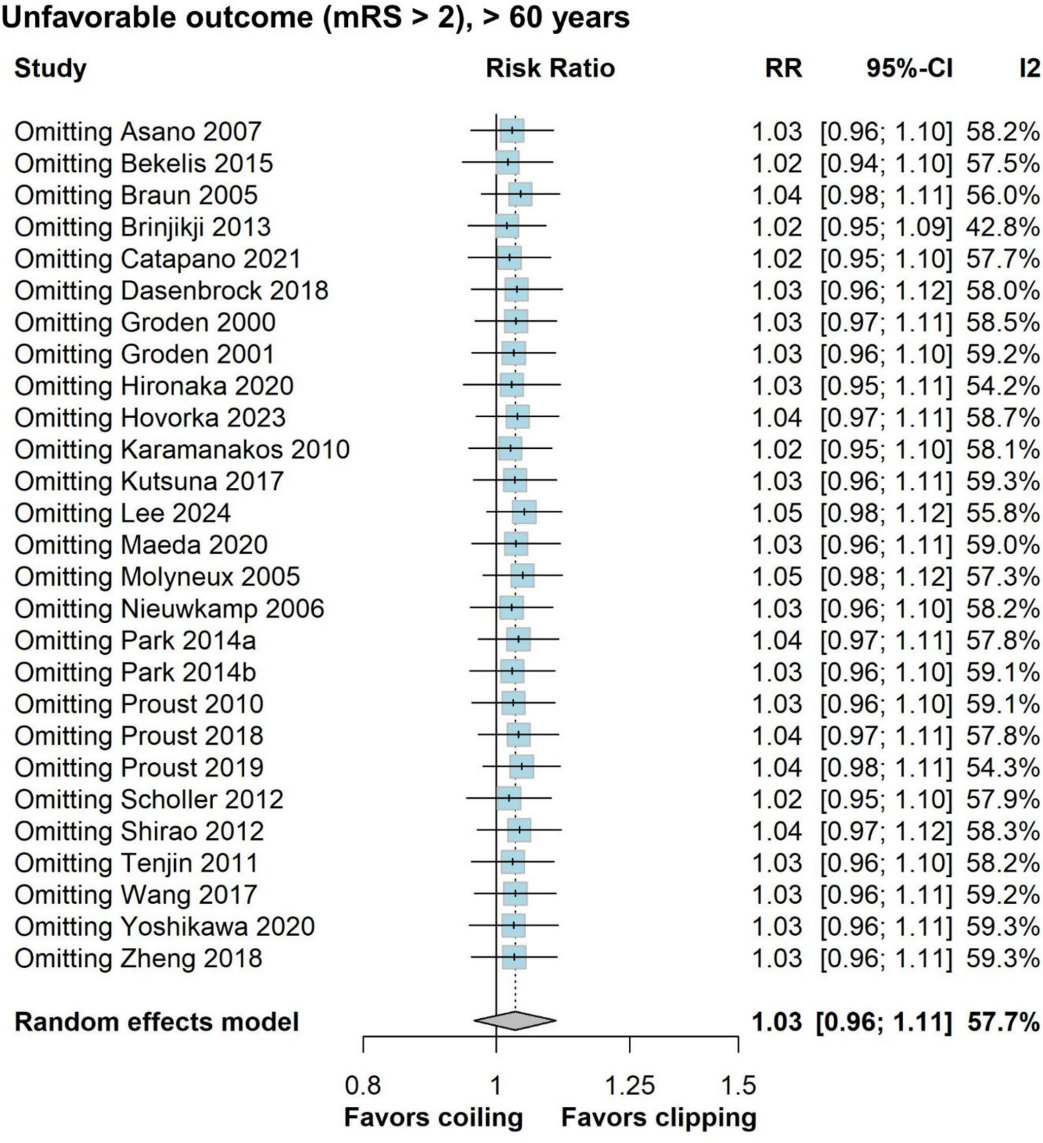
Leave-one-out sensitivity analysis comparing coiling versus clipping for unfavorable outcome (mRS > 2) in patients ≥ 60 years old. RR, risk ratio

**Fig. 4:**
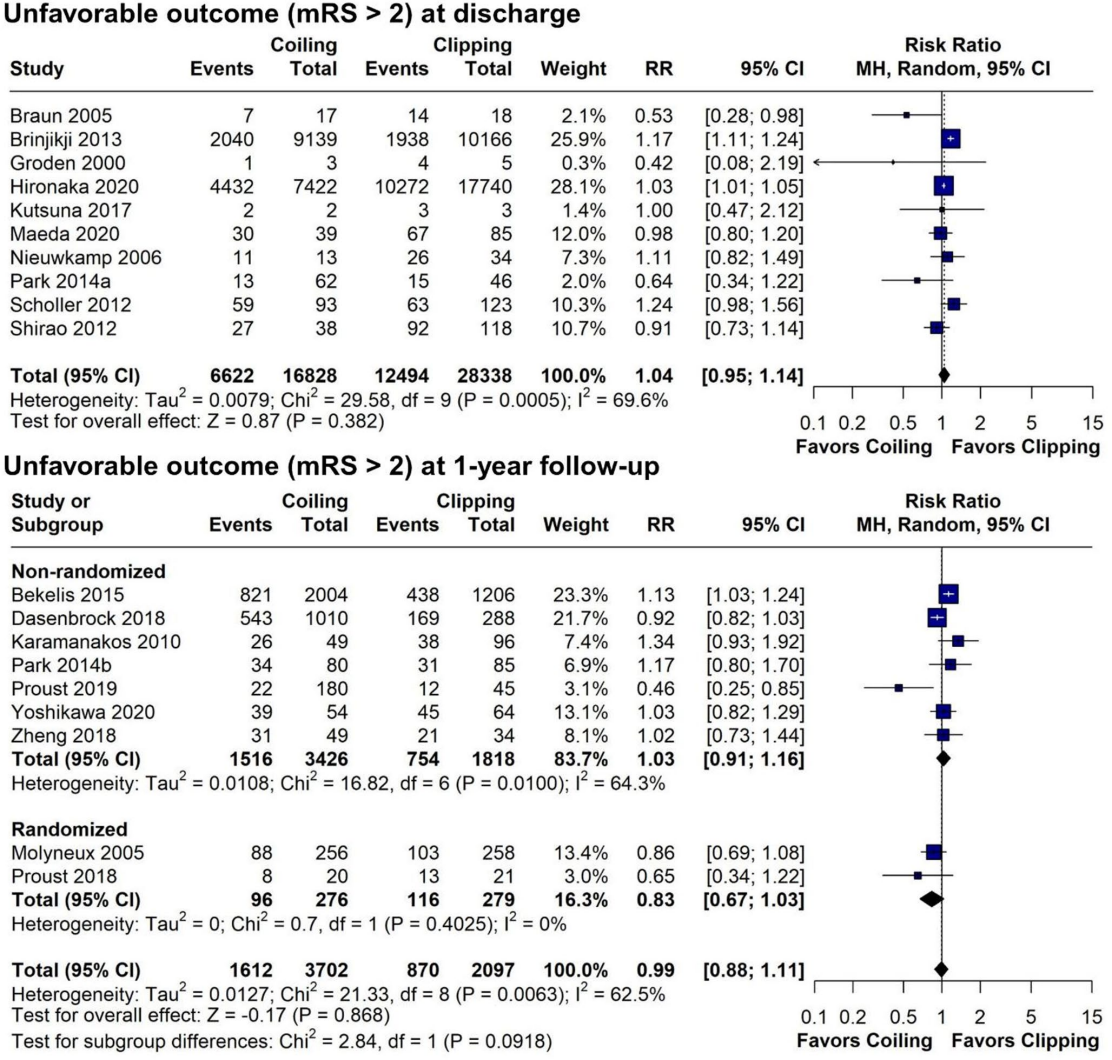
Forests plots comparing coiling versus clipping for unfavorable outcome (mRS > 2) in patients ≥ 60 years old from discharge and to 1-year follow-up. RR, risk ratio

In our primary analysis of 51,415 elderly SAH patients (20,730 in the endovascular coiling group vs. 30,685 in the neurosurgical clipping group) we found no significant difference in unfavorable outcomes at the longest available follow-up (RR = 1.03; 95% CI 0.96–1.11; *p* = 0.369; I^2^ = 57.7%; Fig. 2). Only 3 studies did not report either the mRS or the Glasgow Outcome Scale, and therefore, we used mortality from these studies for the composite outcome [11, 30, 32]. Results remained consistent in the leave-one-out sensitivity analysis (Fig. 3) and across age subgroups: ≥ 70 years (RR = 1.04; 95% CI 0.97–1.11; *p* = 0.256; I^2^ = 39.1%; Supplemental Digital Content 2, Figure S1) and ≥ 80 years-old (RR = 1.04; 95% CI 0.93–1.15; *p* = 0.524; I^2^ = 64.4% (Supplemental Digital Content 2, Figure S2). Additionally, comparison of unfavorable outcomes of all patients aged ≥ 60 years, were consistent from discharge (RR = 1.04; 95% CI 0.95–1.14; *p* = 0.382; I^2^ = 69.6%; Fig. 4) to 1-year follow-up (RR 0.99; 95% CI 0.88–1.11; 0 = 0.868; I^2^ = 62.5%; Fig. 4). At 1-year follow-up, a non-significant trend favored coiling in the RCT subgroup (RR 0.83; 95% CI 0.67–1.03; *p* = 0.4025; I^2^ = 0%). As for potential confounders, posterior aneurysm location (X^2^ = 108.7, df = 1, *p* < 0.01), but not sex (X^2^ = 0.18196, df = 1, *p* = 0.6697), may have an effect in the results, as the posterior circulation group had a greater proportion of unfavorable outcomes. Since only 2 studies reported outcomes for posterior fossa aneurysms in the elderly population, a comparison between treatment groups was not feasible [38, 40].

### Secondary outcomes

#### Mortality rates

**Fig. 5:**
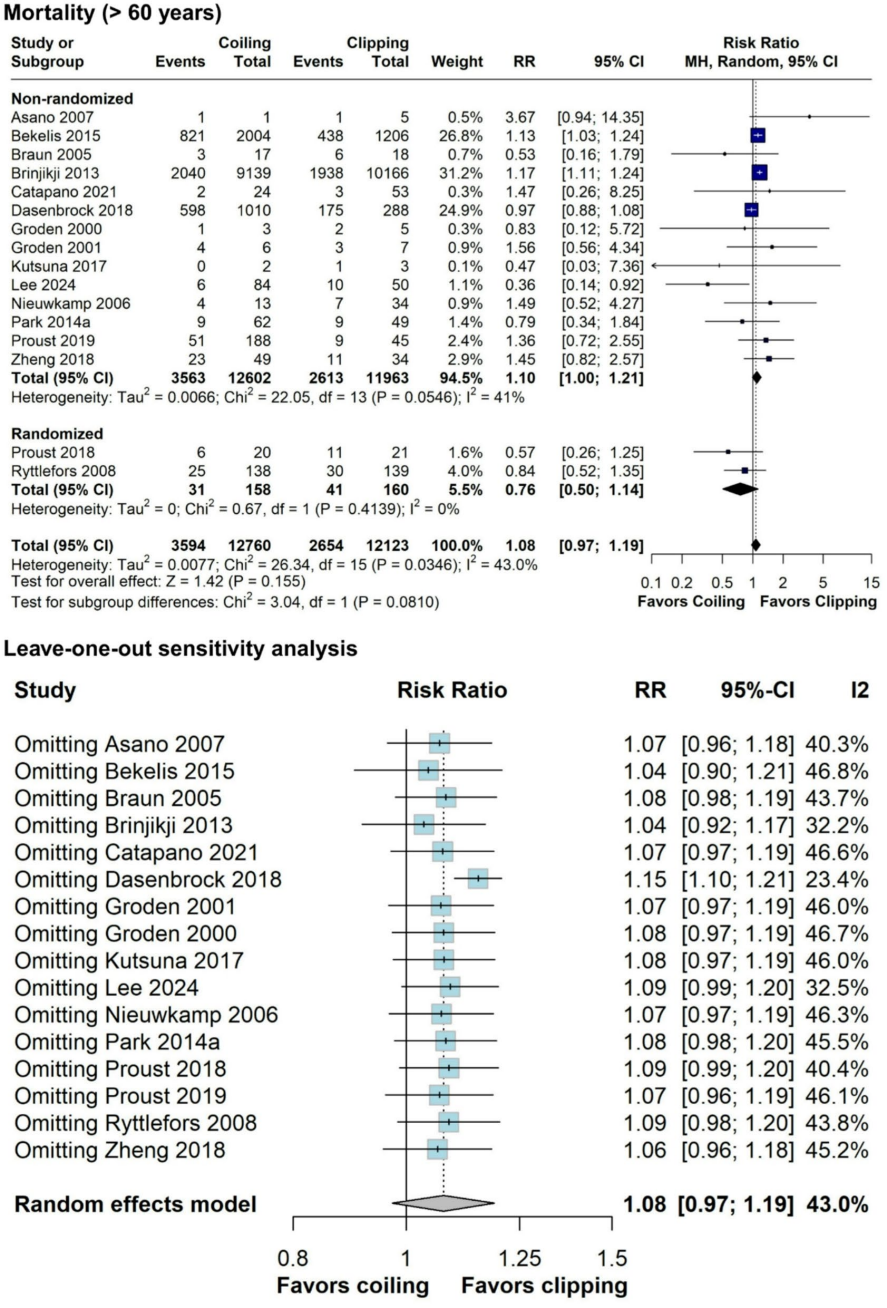
Forest plot comparing coiling versus clipping for mortality in patients ≥ 60 years old and leave-one-out sensitivity analysis. RR, risk ratio

Among SAH patients ≥ 60 years, mortality was comparable between coiling and clipping groups (RR = 1.08; 95% CI 0.97–1.19; *p* = 0.155; I^2^ = 43%; Fig. 5). Interestingly, subgroup analyses of randomized and non-randomized studies revealed opposing directions of effect. The RCTs showed a point estimate favoring coiling (RR = 0.76; 95% CI 0.50–1.14; *p* = 0.4139; I^2^ = 0%), while the observational studies exhibited a trend favoring clipping (RR = 1.10; 95% CI 1.00–1.21; *p* = 0.0546; I^2^ = 41%). Subgroup analysis for patients aged ≥ 70 (Supplemental Digital Content 2, Figure S3) and ≥ 80 years old (Supplemental Digital Content 2, Figure S4) did not have substantial differences from the main analysis. In the leave-one-out sensitivity analysis, the results remained largely stable, except when the study by Dasenbrock et al. was removed (Fig. 5) [32]. The subgroup analysis of adjusted estimates and RCTs for mortality in individuals aged over 60 years was not statistically significant (Supplemental Digital Content 2, Figure S5).

#### Favorable outcome (mRS 0–2), rebleeding, and hospital length of stay

**Fig. 6:**
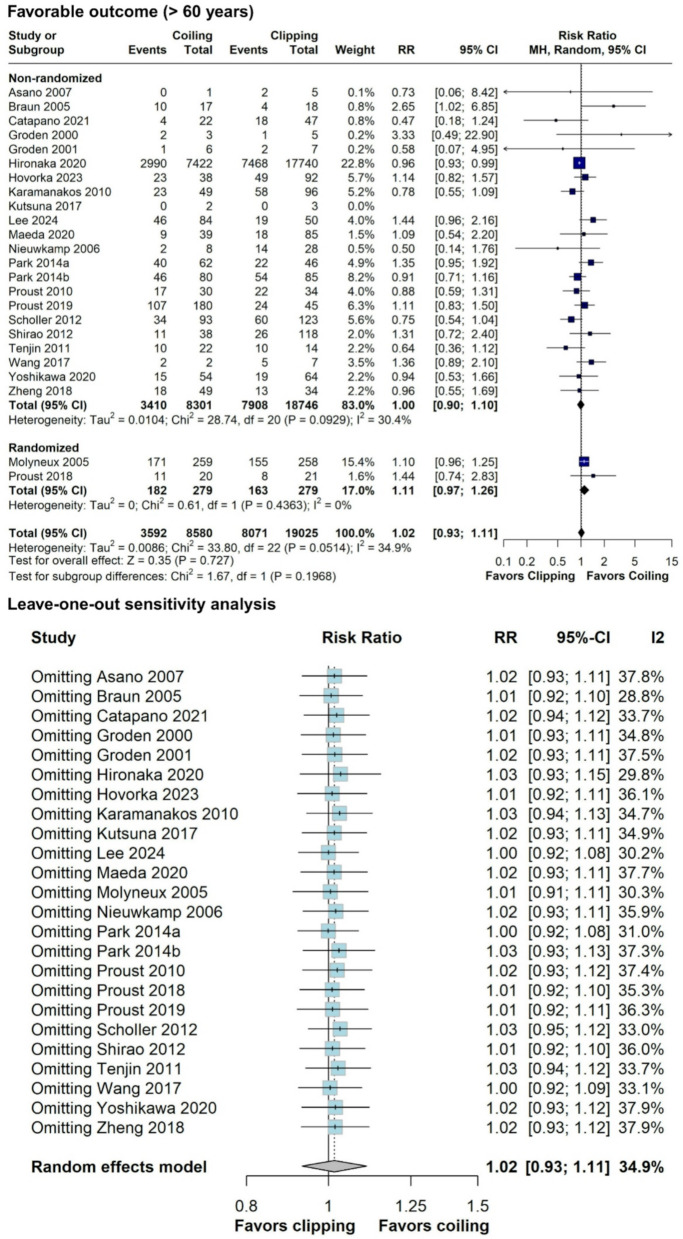
Forest plot comparing coiling versus clipping for favorable outcome (mRS 0–2) in patients ≥ 60 years old and leave-one-out sensitivity analysis. RR, risk ratio

**Fig. 7:**
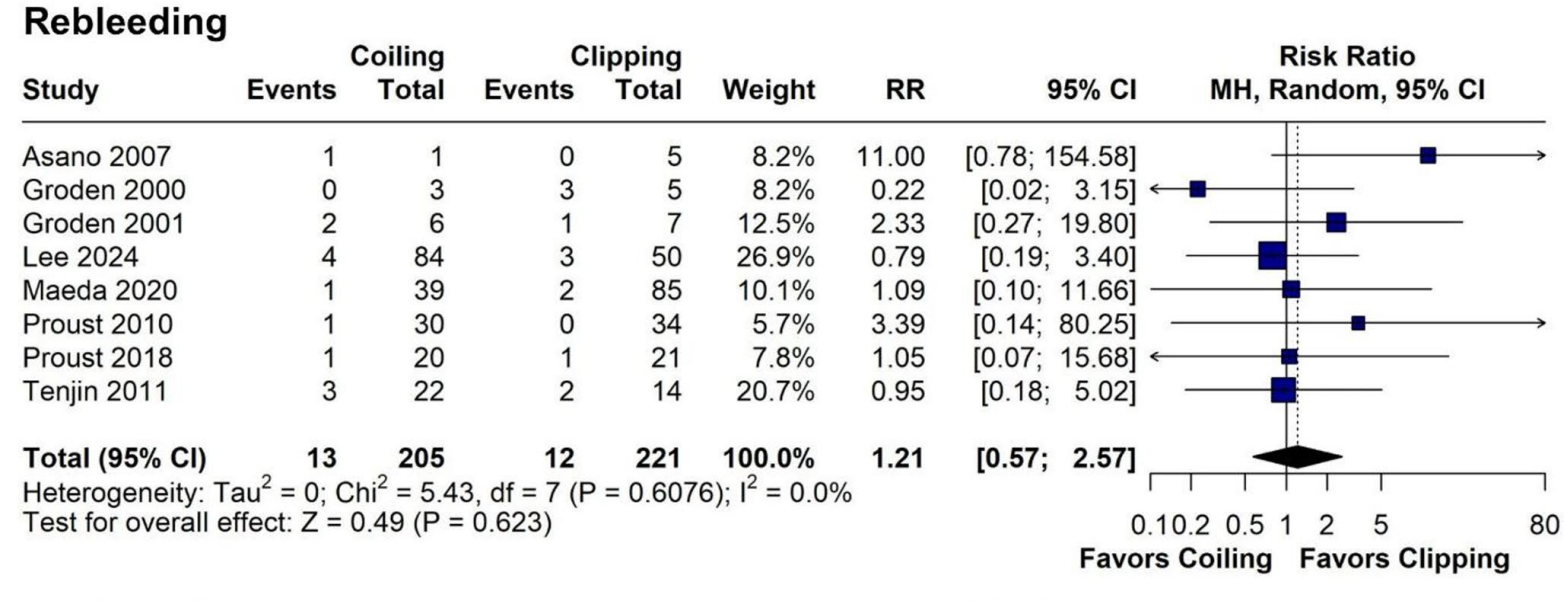
Forest plot comparing coiling versus clipping for rebleeding in patients ≥ 60 years old. RR, risk ratio

**Fig. 8:**
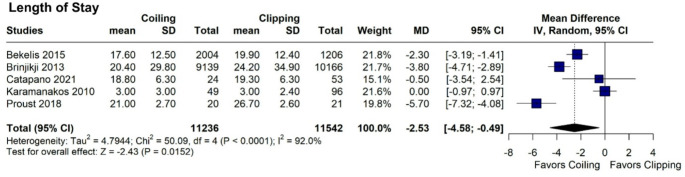
Forest plot comparing coiling versus clipping for hospital length of stay in patients ≥ 60 years old. RR, risk ratio

Both endovascular coiling and neurosurgical clipping showed similar rates of favorable outcomes (mRS 0–2; RR = 1.02; 95% CI = 0.93–1.11; *p* = 0.727; I^2^ = 34.9%; Fig. 6), with consistent results in sensitivity analysis, indicating robustness (Fig. 6). Subgroup analysis based on randomization status revealed trends consistent with those observed for mortality rates (Fig. 6). Pooled favorable outcome (mRS 0–2) rate was 41.8% (*N* = 11,318/27,047). Out of 24 studies, only 2 studies considered good outcome an mRS 0–3 [42, 47]. Rebleeding rates were also comparable (RR = 1.21; 95% CI 0.57–2.57; *p* = 0.623; I^2^ = 0%; Fig. 7). However, endovascular coiling was associated with a significant reduction in hospital length of stay by an average of 2.53 days (95% CI -4.58 - -0.49; *p* = 0.0152; Fig. 8), albeit with high heterogeneity (I^2^ = 92%).

### Publication bias and confidence in the evidence

Visual inspection of the funnel plots (Supplemental Digital Content 2, Figure S6, Figure S7, and Figure S8) and the Egger’s test for the primary outcome (mRS > 2 or mortality) (intercept = -0.202; 95% CI -0.89–0.48; t -0.577; *p* = 0.569), mortality (intercept = -0.405; 95% CI -1.19–0.38; t = -1.018; *p* = 0.325), and favorable outcome (mRS 0–2) (intercept = 0.319; 95% CI -0.27–0.9; t = 1.063; *p* = 0.299) revealed no significant publication bias. Using the GRADE framework, the overall certainty in the evidence was assessed as very low [23], primarily due to the dominance of non-randomized studies, moderate risk of bias across included studies, and substantial heterogeneity (I^2^ = 57.7%) in the main analysis (Supplemental Digital Content 1, Table S4).

## Discussion

This systematic review and meta-analysis compared endovascular coiling versus surgical clipping for aneurysmal SAH in elderly patients, pooling data from 27 studies (*n* = 51,415). No significant differences were observed between the two modalities in composite unfavorable outcomes (RR = 1.03; *p* = 0.369), favorable outcomes (RR = 1.02; *p* = 0.727), mortality (RR = 1.08; *p* = 0.155), or rebleeding (RR = 1.21; *p* = 0.623). Coiling, however, was associated with shorter hospital stays (MD = 2.53; *p* = 0.0152), albeit with high heterogeneity (I^2^ = 92%), likely due to variation in healthcare systems, discharge practices, and study characteristics, including single-center designs across different countries and broad inclusion periods (1983–2012).

These findings were largely robust to leave-one-out sensitivity analysis, except mortality, which demonstrated variability, particularly upon exclusion of the largest study (Dasenbrock et al.). Mortality appears vulnerable to bias and heterogeneity and should be interpreted cautiously. Subgroup analyses stratified by age and follow-up duration yielded consistent results. Interestingly, observational studies tended to favor clipping, while RCTs favored coiling, underscoring the influence of study design and potential confounding in non-randomized data. Indeed, the overall certainty in the evidence was assessed as very low by the GRADE framework.

The ISAT trial demonstrated superior outcomes with coiling over clipping, including a 24% relative risk reduction in death or dependency at one year, albeit with slightly increased rebleeding risk [25]. These benefits persisted at 10-year follow-up [9]. Notably, newer endovascular devices such as intrasaccular WEBs—unavailable during ISAT—may further enhance the net benefit, as they allow treatment of broad-based aneurysms with low complication rates and without the need for antiplatelet therapy [50]. In contrast, the BRAT trial reported no significant difference between modalities in anterior circulation aneurysms, although posterior circulation aneurysms showed better outcomes with coiling [51].

Microsurgery remains a valuable technique for challenging aneurysms not amenable to endovascular treatment, like basilar artery perforator aneurysms [52]. Additionally, recovery from oculomotor nerve palsy associated with posterior communicating artery aneurysms seems to be higher after microsurgery [53]. However, the technical challenges of clipping posterior circulation aneurysms—including their deep location, complex vascular anatomy, and frequent irregular morphology—likely contribute to outcome disparities observed between clipping and coiling [54, 55]. These anatomical constraints introduce a systematic bias in observational studies, as high-risk posterior fossa aneurysms are often preferentially treated with endovascular methods, thereby underrepresenting them in surgical cohorts [56]. This bias was evident in a meta-analysis showing that coiling appeared superior overall, but when posterior circulation aneurysms were excluded, outcome differences between treatments disappeared (*p* = 0.32) [57].

Institutional expertise and procedural volume also influence outcomes. Single-center studies reflect local practices and experience, and a well-documented volume-outcome relationship in aneurysmal SAH shows that higher caseloads are associated with lower mortality [58, 59]. These factors contribute to variation in reported outcomes and may mask true treatment effects in observational data. For instance, a recent meta-analysis of RCTs demonstrated a persistent reduction in poor outcomes at one year with coiling, even in worst-case scenario analyses [60]. This effect may be obscured in observational data due to selection bias, with elderly and functionally impaired patients (e.g., baseline mRS 3–5) more frequently triaged to endovascular therapy, which is associated with better functional outcomes for poor grade patients [61]. Our sensitivity analysis supports this concern; when excluding Dasenbrock et al.‘s large observational series (*n* = 1,298) where 77% of patients had good-grade SAH (NIS-SSS < 7) [62], the overall results shifted toward favoring clipping [32].

Furthermore, most observational studies failed to adjust for baseline severity using validated prognostic scales (e.g., WFNS [63], Fisher [64], modified Fisher [65], VASOGRADE [66]), limiting risk stratification and confounding control in our pooled analysis. Our analysis found no significant difference in rebleeding rates between endovascular coiling and surgical clipping, a finding that contrasts with randomized clinical trials. While some studies report a higher rebleeding risk with coiling, the absolute risk remains low (2–3% in most series) [9, 51]. However, these findings should be tempered by the limited long-term follow-up in our dataset (12–180 months), as rebleeding risks may diverge beyond the perioperative period.

Finally, coiling was associated with shorter hospital stays, consistent with its minimally invasive nature and prior studies linking it to fewer in-hospital complications [67] and accelerated postoperative recovery compared to surgical clipping, attributable to its minimally invasive approach [67, 68]. This corroborates that while coiling incurs higher procedural costs than clipping, it may offset economic expenditures through shorter inpatient stays [69].

This meta-analysis synthesizes data from 27 international studies encompassing 51,415 patients, enhancing the generalizability across diverse populations. Our methodology adhered to the highest standards, including prospective PROSPERO registration and strict adherence to PRISMA guidelines and Cochrane Handbook recommendations. Importantly, we addressed a critical knowledge gap by focusing on elderly patients, a population for whom evidence derives primarily from observational studies with inherent selection biases that reflect institutional practices rather than true treatment effects. Given the logistical and ethical barriers to RCTs in this group, our study provides the most comprehensive evidence currently achievable.

However, several limitations warrant consideration. The predominance of unadjusted observational data introduces a high risk of confounding related to baseline clinical status and comorbidities. To address this, we conducted a subgroup analysis for mortality, including the only observational study with adjusted estimates alongside the two RCTs [11, 24, 48], aiming to reduce bias from unadjusted comparisons. Our primary outcome combined mortality and mRS, which may introduce bias; however, this composite outcome was chosen to maximize study inclusion and minimize data exclusion. Key outcomes for the elderly cohort, such as quality of life [13, 24] and delayed cerebral ischemia [39, 48], were reported in too few studies for meaningful analyses. Furthermore, meta-regression and subgroup analyses for known confounders (e.g., WFNS grade, baseline mRS, aneurysm location, posterior aneurysm circulation) were not feasible due to limited reporting. The lack of data on timing of rebleeding and mortality also limited our ability to assess temporal outcome patterns [33, 34, 38, 39, 41, 49].

## Conclusion

This meta-analysis of observational and randomized studies comparing endovascular coiling and microsurgical clipping for aneurysmal SAH in elderly patients has two main findings: (1) no significant differences in composite unfavorable outcomes, mortality, favorable outcomes, or rebleeding (2) coiling resulted in shorter hospital stays. However, most evidence comes from unadjusted observational studies, reflected in the overall very low GRADE rating. These results should be interpreted with caution and validated through prospective, multicenter cohorts that incorporate modern endovascular techniques and robust risk stratification, as RCTs in this population would be both ethically and logistically impractical.

## Electronic supplementary material

Below is the link to the electronic supplementary material.

Supplementary Material 1

Supplementary Material 2

## Data Availability

No datasets were generated or analysed during the current study.

## Abbreviations

BRAT: Barrow Ruptured Aneurysm Trial
CI: Confidence Interval
GRADE: Grading of Recommendations Assessment, Development, and Evaluation
IQR: Interquartile range
ISAT: International subarachnoid aneurysm trial
MD: Mean Difference
mRS: Modified Rankin Scale
NIS: SSS-Nationwide Inpatient Sample Subarachnoid Hemorrhage Severity Score
NOS: Newcastle Ottawa Scale
PICOTS: Population, Intervention, Comparison, Outcome, Timing, and Setting
PRISMA: Preferred Reporting Items for Systematic Review and Meta-Analysis
PROSPERO: International Prospective Register of Systematic Reviews
RCT: Randomized Controlled Trial
RoB 2: Revised Cochrane risk-of-bias tool
RR: Risk Ratio
SAH: Subarachnoid hemorrhage
SD: Standard Deviation
WFNS: World Federation of Neurological Surgeons
