# Supplementary material for: Comparative outcomes of coil embolization and surgical clipping in elderly patients with subarachnoid hemorrhage: a systematic review and meta-analysis
Source: Neurosurg Rev. 2025 Aug 4;48(1):587. doi: 10.1007/s10143-025-03713-9 (PMC12318887; doi:10.1007/s10143-025-03713-9)
Supplement: Supplementary file 1 — Supplementary Material 1 [file 10143_2025_3713_MOESM1_ESM.pdf]

Supplemental Digital Content 1.
Tables and search strategy

Article title: Comparative Outcomes of Coil Embolization and Surgical Clipping in Elderly Patients with Subarachnoid Hemorrhage: A Systematic Review and Meta-Analysis
Journal name: Neurosurgical Review
Author names: Yohanna Idsabella Rossi[1]; Gabriel Bolner[1], Jonathan Costa Dall'Acqua[1]; Fabiana Dolovitsch de Oliveira[1]; Lucas Vincenzi Zacaria[1]; Taís Luise Denicol[1], MD; Michel Frudit[2,3], MD, PhD; Natália Vasconcellos de Oliveira Souza, MD, MSc[3,4].

1 - Federal University of Health Sciences of Porto Alegre, Porto Alegre, Rio Grande do Sul, Brazil.

2 – University of São Paulo, Intervention Neuroradiology and Neurosurgery Department, São Paulo, Brazil.

3 - Albert Einstein Hospital, Neurology and Intervention Neuroradiology Department, São Paulo, Brazil.

4 – Department of Neurocritical Care, Mayo Clinic, Jacksonville, Florida, USA

Corresponding author: Natália Vasconcellos de Oliveira Souza
Email: dranataliavasconcellos@gmail.com. Souza.Natalia@mayo.edu

1. Search strategy

"subarachnoid hemorrhage" AND (coiling OR endovascular OR embolization) AND clipping AND (older OR elder OR elderly OR >65 OR "> 65" OR >60 OR "> 60" OR "> 70" OR >70 OR >75 OR ">75" OR "> 80" OR >80 OR >85 OR "> 85" OR old)

2. **Table S2:** Risk of Bias (Rob) 2 tool for randomized clinical trials comparing clipping versus coiling in the elderly population

Study	Domain 1	Domain 2	Domain 3	Domain 4	Domain 5	Rating
**Molyneux et al. (2005)**	Some concerns	Some concerns	Low risk	Some concerns	Low risk	**Some concerns**
**Proust et al. (2018)**	Some concerns	Low risk	Low risk	Some concerns	Low risk	**Some concerns**
Domains. 1: Risk of bias arising from the randomization process. 2: Risk of bias due to deviations from the intended interventions. 3: Missing outcome data. 4: Risk of bias in measurement of the outcome. 5: Risk of bias in selection of the reported result.						

3.**Table S3:** Risk of bias assessment for cohort studies comparing clipping versus coiling in the elderly population – Newcastle Ottawa Scale (NOS)

Study	Selection			Comparability		Outcome/Exposure		Rating
	**Representativeness of the exposed cohort**	**Ascertainment of exposure**	**Outcome of interest not present at start of study**	**Controls for variable clinical elements of aSAH**	**Controls for other factors (e.g., sex, age)**	**Ascertainment of exposure**	**Adequacy of follow up**	
**Asano et al. (2007)**	Yes	Yes	No	No	No	Yes	Yes	**Moderate** *
**Bekelis et al. (2015)**	No	Yes	No	No	No	Yes	Yes	**Moderate** *
**Braun et al. (2005)**	Yes	Yes	No	No	No	Yes	Yes	**Moderate** *

**Catapano et al. (2021)**	Yes	Yes	No	No	Yes	Yes	Yes	**Low***
**Dasenbrock et al. (2019)**	Yes	Yes	No	No	No	Yes	Yes	**Moderate***
**Groden et al. (2000)**	Yes	Yes	No	No	No	Yes	Yes	**Moderate***
**Groden et al. (2001)**	No	Yes	No	No	Yes	Yes	Yes	**Low***
**Hironaka et al. (2020)**	Yes	Yes	No	No	No	Yes	No	**Moderate***
**Hovorka et al. (2023)**	Yes	Yes	No	No	No	Yes	Yes	**Moderate***
**Karamanakos et al. (2010)**	Yes	Yes	No	No	Yes	Yes	No	**Moderate***
**Kutsuna et al. (2017)**	No	Yes	No	No	Yes	Yes	Yes	**Moderate***

**Lee et al. (2024)**	Yes	Yes	No	No	No	Yes	Yes	**Moderate***
**Maeda et al. (2020)**	Yes	Yes	No	No	No	Yes	No	**Moderate***
**Nieuwkamp et al. (2006)**	Yes	Yes	No	No	Yes	Yes	Yes	**Low***
**Park et al. (2014)**	Yes	Yes	No	No	No	Yes	No	**Moderate***
**Park (critical age) et al. (2018)**	No	Yes	No	No	Yes	Yes	Yes	**Moderate***
**Proust et al. (2010)**	Yes	Yes	No	No	No	Yes	Yes	**Moderate***
**Proust et al. (2019)**	Yes	Yes	No	No	No	Yes	Yes	**Moderate***
**Scholler et al. (2012)**	Yes	Yes	No	No	No	Yes	Yes	**Low***

**Shirao et al. (2012)**	Yes	Yes	No	No	Yes	Yes	No	**Moderate ***
**Tenjin et al. (2011)**	No	Yes	No	No	No	Yes	Yes	**Moderate ***
**Wang et al. (2017)**	Yes	Yes	No	No	No	Yes	Yes	**Moderate ***
**Yoshikawa et al. (2020)**	Yes	Yes	No	No	No	Yes	Yes	**Moderate ***
**Zheng et al. (2018)**	Yes	Yes	No	No	No	Yes	No	**Moderate ***
**aSAH: aneurysmal Subarachnoid Hemorrhage**								

4. **Table S4.** GRADE strategy for assessing certainty of evidence

Question: Coil embolization compared to surgical clipping for Unfavorable outcome
Setting: What are the comparative effectiveness of coil embolization versus surgical clipping for elderly patients with aneurysmal subarachnoid hemorrhage (aSAH)?

Bibliography:

Certainty assessment							№ of patients		Effect		Certainty	Importance
**№ of studies**	**Study design**	**Risk of bias**	**Inconsistency**	**Indirectness**	**Imprecision**	**Other considerations**	**coil embolization**	**surgical clipping**	**Relative (95% CI)**	**Absolute (95% CI)**		
**Unfavorable outcome**												
25	non-randomised studies	serious[a]	serious[b]	not serious	not serious	all plausible residual confounding would reduce the demonstrated effect	19263/49540 (38.9%)	30277/49540 (61.1%)	**RR 1.03** (0.96 to 1.11)	**18 more per 1.000** (from 24 fewer to 67 more)	⊕◯◯◯ Very low[a,b]	IMPORTANT

CI: confidence interval; **RR:** risk ratio

Explanations

a. Most included studies had a Moderate risk of bias

b. High Heterogeneity
