# Supplementary material for: Comparative outcomes of coil embolization and surgical clipping in elderly patients with subarachnoid hemorrhage: a systematic review and meta-analysis
Source: Neurosurg Rev. 2025 Aug 4;48(1):587. doi: 10.1007/s10143-025-03713-9 (PMC12318887; doi:10.1007/s10143-025-03713-9)

Supplemental Digital Content 2
Forest Plots

Article title: Comparative Outcomes of Coil Embolization and Surgical Clipping in Elderly Patients with Subarachnoid Hemorrhage: A Systematic Review and Meta-Analysis

Journal name: Neurosurgical Review

Author names: Yohanna Idsabella Rossi[1]; Gabriel Bolner[1], Jonathan Costa Dall'Acqua[1]; Fabiana Dolovitsch de Oliveira[1]; Lucas Vincenzi Zacaria[1]; Taís Luise Denicol[1], MD; Michel Frudit[2,3], MD, PhD; Natália Vasconcellos de Oliveira Souza, MD, MSc[3,4].

1 - Federal University of Health Sciences of Porto Alegre, Porto Alegre, Rio Grande do Sul, Brazil.

2 – University of São Paulo, Intervention Neuroradiology and Neurosurgery Department, São Paulo, Brazil.

3 - Albert Einstein Hospital, Neurology and Intervention Neuroradiology Department, São Paulo, Brazil.

4 – Department of Neurocritical Care, Mayo Clinic, Jacksonville, Florida, USA

Corresponding author: Natália Vasconcellos de Oliveira Souza

Email:

dranataliavasconcellos@gmail.com.

Souza.Natalia@mayo.edu

Forest Plots

Figure S1. Forest plot comparing coiling versus clipping for unfavorable outcome (mRS > 2) in patients ≥ 70 years old. RR, risk ratio.

Study or Subgroup	Coiling Events	Coiling Total	Clipping Events	Clipping Total	Weight	RR	95% CI
**Non-randomized**							
Asano 2007	1	1	3	5	1.1%	1.57	[0.84; 2.96]
Brinjikji 2013	608	2023	342	1322	17.2%	1.16	[1.04; 1.30]
Dasenbrock 2018	598	1010	175	288	18.2%	0.97	[0.88; 1.08]
Groden 2000	1	2	2	2	0.5%	0.60	[0.22; 1.65]
Groden 2001	2	3	2	3	0.4%	1.00	[0.32; 3.10]
Hironaka 2020	3230	4649	6513	9334	31.2%	1.00	[0.97; 1.02]
Karamanakos 2010	26	49	38	96	3.2%	1.34	[0.93; 1.92]
Kutsuna 2017	2	2	3	3	0.8%	1.00	[0.47; 2.12]
Maeda 2020	30	39	67	85	8.3%	0.98	[0.80; 1.20]
Nieuwkamp 2006	6	8	14	28	1.5%	1.50	[0.87; 2.59]
Park 2014b	34	80	31	85	3.0%	1.17	[0.80; 1.70]
Proust 2010	13	30	12	34	1.2%	1.23	[0.67; 2.26]
Proust 2019	22	180	12	45	1.2%	0.46	[0.25; 0.85]
Tenjin 2011	12	22	4	14	0.6%	1.91	[0.77; 4.75]
Yoshikawa 2020	39	54	45	64	7.0%	1.03	[0.82; 1.29]
**Total (95% CI)**	**4624**	**8152**	**7263**	**11408**	**95.2%**	**1.04**	**[0.97; 1.12]**
Heterogeneity: $Tau^2 = 0.0041$; $Chi^2 = 23.79$, df = 14 (P = 0.0486); $I^2 = 41.2\%$							
**Randomized**							
Molyneux 2005	34	61	31	64	3.6%	1.15	[0.82; 1.61]
Proust 2018	8	20	13	21	1.1%	0.65	[0.34; 1.22]
**Total (95% CI)**	**42**	**81**	**44**	**85**	**4.8%**	**0.92**	**[0.53; 1.60]**
Heterogeneity: $Tau^2 = 0.0995$; $Chi^2 = 2.49$, df = 1 (P = 0.1148); $I^2 = 59.8\%$							
**Total (95% CI)**	**4666**	**8233**	**7307**	**11493**	**100.0%**	**1.04**	**[0.97; 1.11]**

Heterogeneity: $Tau^2 = 0.0038$; $Chi^2 = 26.28$, df = 16 (P = 0.0502); $I^2 = 39.1\%$
Test for overall effect: Z = 1.13 (P = 0.256)
Test for subgroup differences: $Chi^2 = 0.20$, df = 1 (P = 0.6571)

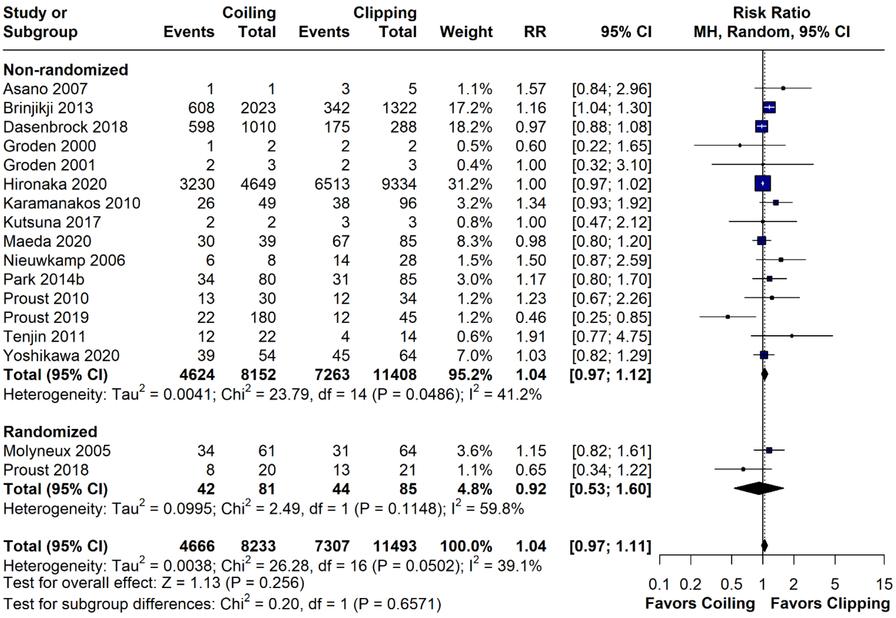

Figure S2. Forest plot comparing coiling versus clipping for unfavorable outcome (mRS > 2) in patients ≥ 80 years old. RR, risk ratio.

Study	Coiling Events	Coiling Total	Clipping Events	Clipping Total	Weight	RR	95% CI
Asano 2007	1	1	3	5	2.7%	1.57	[0.84; 2.96]
Brinjikji 2013	608	2023	342	1322	27.7%	1.16	[1.04; 1.30]
Dasenbrock 2018	598	1010	175	288	28.8%	0.97	[0.88; 1.08]
Hironaka 2020	1602	2019	2303	2819	38.9%	0.97	[0.94; 1.00]
Kutsuna 2017	2	2	3	3	1.9%	1.00	[0.47; 2.12]
**Total (95% CI)**	**2811**	**5055**	**2826**	**4437**	**100.0%**	**1.04**	**[0.93; 1.15]**

Heterogeneity: $Tau^2 = 0.0075$; $Chi^2 = 11.25$, df = 4 (P = 0.0239); $I^2 = 64.4\%$
Test for overall effect: Z = 0.64 (P = 0.524)

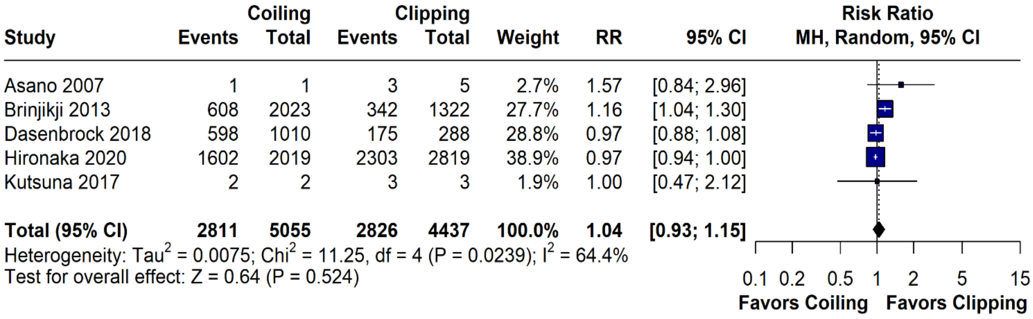

Figure S3. Forest plot comparing coiling versus clipping for all-cause mortality in patients ≥ 70 years old. RR, risk ratio.

Study	Coiling Events	Coiling Total	Clipping Events	Clipping Total	Weight	RR	95% CI
Asano 2007	1	1	1	5	1.2%	3.67	[0.94; 14.35]
Brinjikji 2013	608	2023	342	1322	41.7%	1.16	[1.04; 1.30]
Dasenbrock 2018	598	1010	175	288	42.9%	0.97	[0.88; 1.08]
Groden 2000	1	2	2	2	2.2%	0.60	[0.22; 1.65]
Groden 2001	2	3	1	3	0.7%	2.00	[0.33; 11.97]
Kutsuna 2017	0	2	1	3	0.3%	0.47	[0.03; 7.36]
Nieuwkamp 2006	4	13	7	34	2.1%	1.49	[0.52; 4.27]
Proust 2018	6	20	11	21	3.6%	0.57	[0.26; 1.25]
Proust 2019	51	188	9	45	5.3%	1.36	[0.72; 2.55]
**Total (95% CI)**	**1271**	**3262**	**549**	**1723**	**100.0%**	**1.07**	**[0.91; 1.24]**

Heterogeneity: $Tau^2 = 0.0113$; $Chi^2 = 13.54$, df = 8 (P = 0.0945); $I^2 = 40.9\%$
Test for overall effect: Z = 0.81 (P = 0.417)

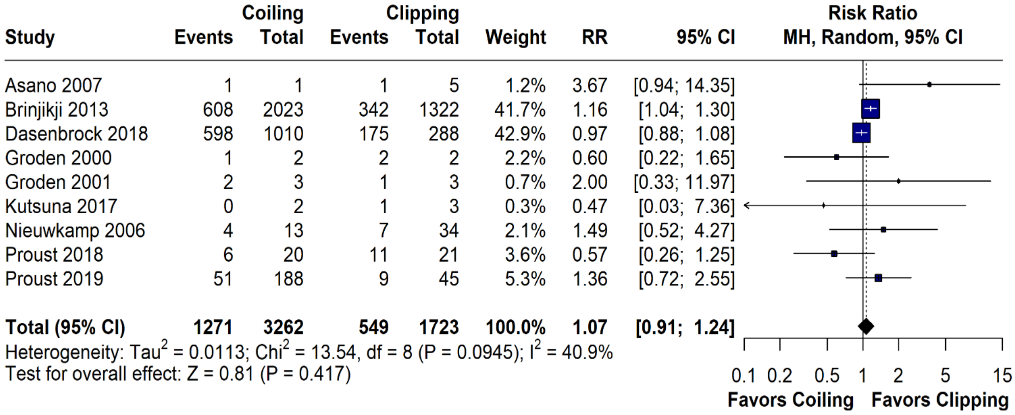

Figure S4. Forest plot comparing coiling versus clipping for all-cause mortality in patients ≥ 80 years old. RR, risk ratio.

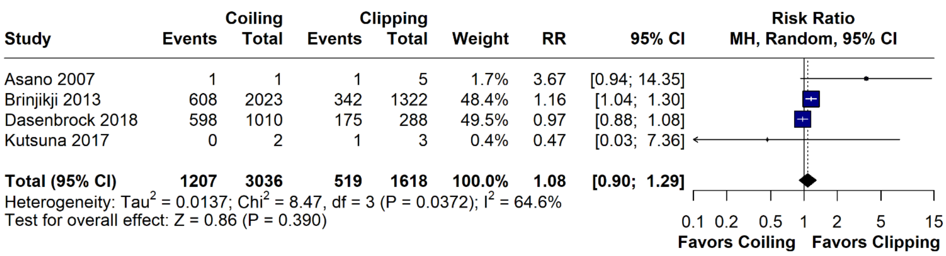

Figure S5. Subgroup analysis of adjusted estimates and randomized clinical trials for mortality in individuals over 60 years.

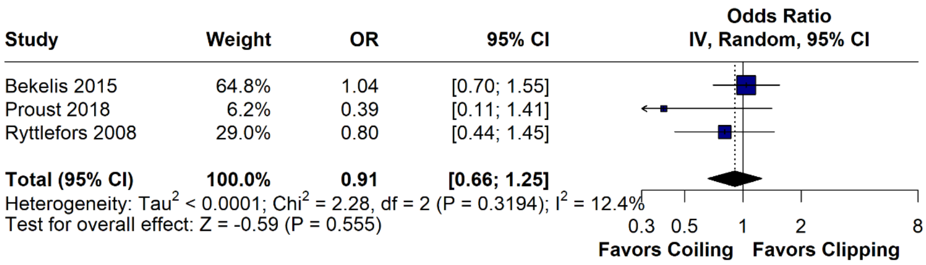

Figure S6. Funnel plot of unfavorable outcomes (mRS > 2 or mortality) in patients ≥ 60 years old.

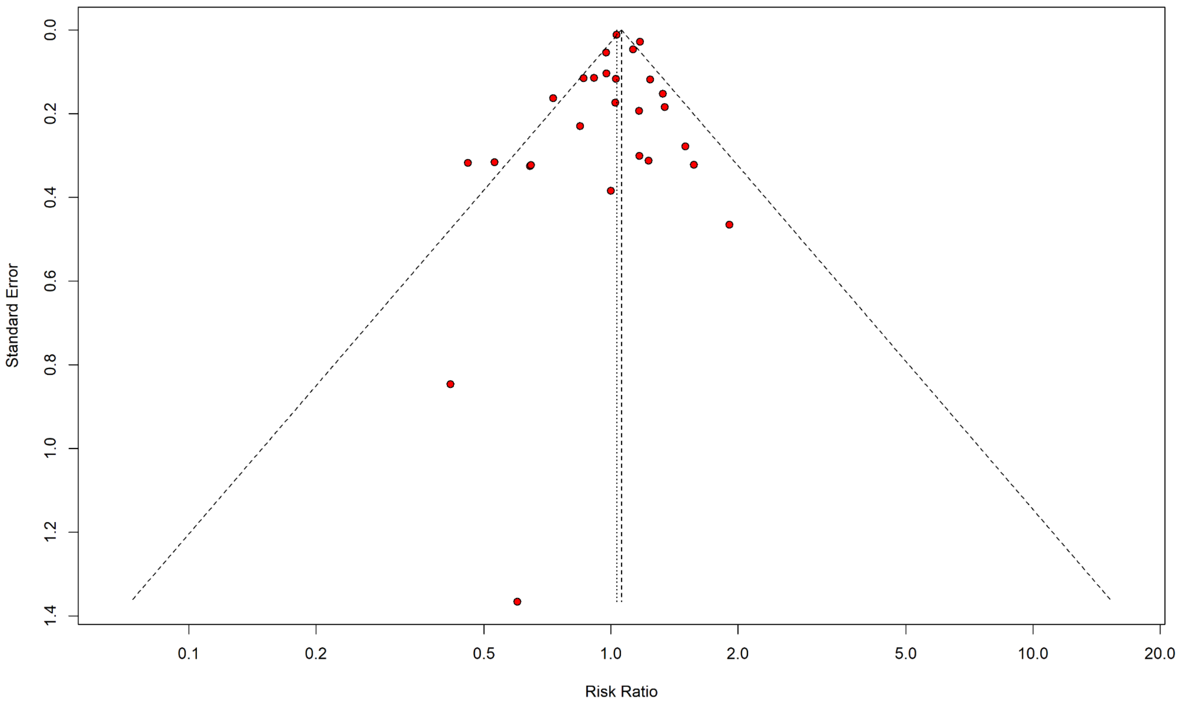

Figure S7. Funnel plot of mortality in patients ≥ 60 years old.

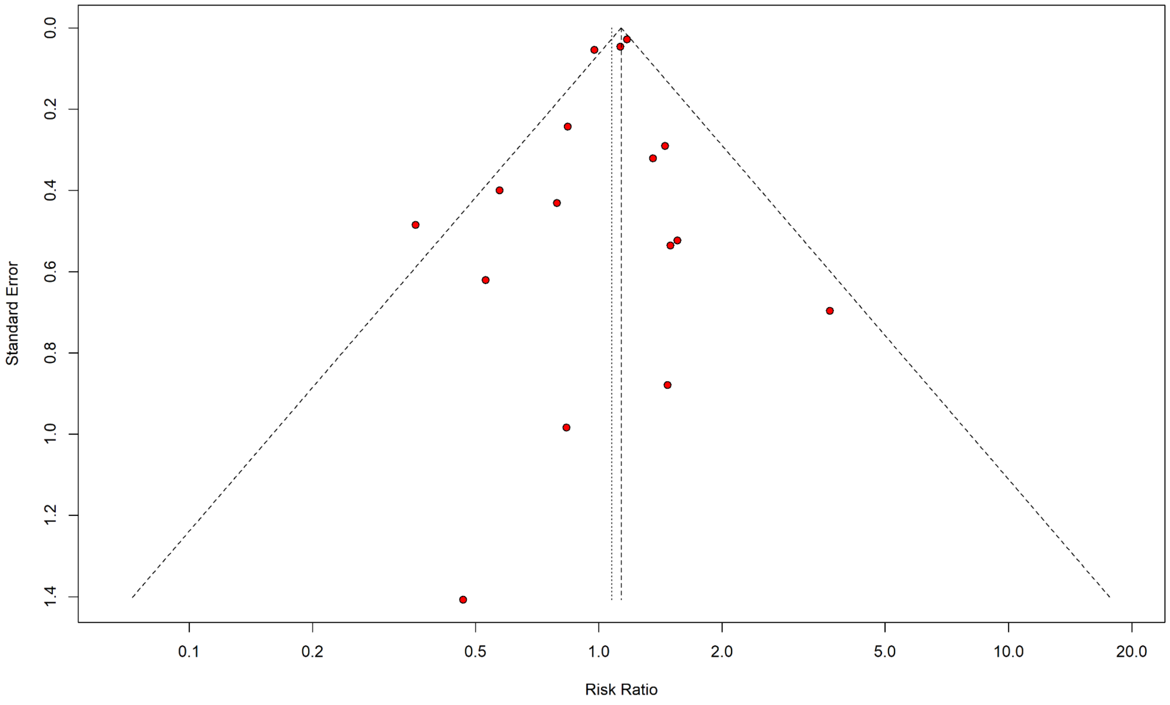

Figure S8. Funnel plot of favorable outcome (mRS 0-2) in patients ≥ 60 years old.

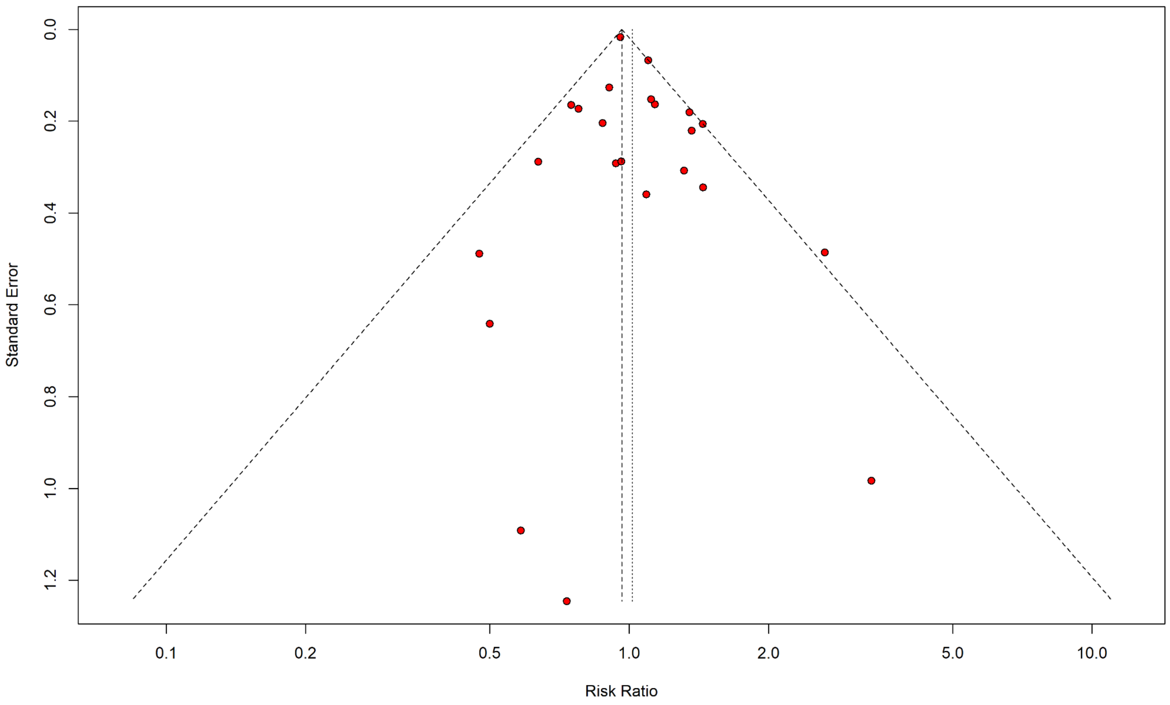

Supplement: Supplementary file 2 — Supplementary Material 2 [file 10143_2025_3713_MOESM2_ESM.pdf]
